# Gulf War Illness: Unifying Hypothesis for a Continuing Health Problem

**DOI:** 10.3390/ijerph16010111

**Published:** 2019-01-03

**Authors:** Anthony R. Mawson, Ashley M. Croft

**Affiliations:** 1 Department of Epidemiology and Biostatistics, School of Public Health, Jackson State University, Jackson, MS 39213, USA; 2 School of Health Sciences and Social Work, University of Portsmouth, Portsmouth P01 2DT, UK; Ashley.croft@myport.ac.uk

**Keywords:** Gulf War Illness, musculoskeletal pain, fatigue, headache, cognition, veterans, risk factors, pathogenesis, vaccines, chemicals, exposures, retinoids

## Abstract

An estimated 25%–32% of veterans of the 1991 Gulf War continue to experience multiple unexplained health problems known as Gulf War Illness (GWI). GWI encompasses chronic pain, musculoskeletal weakness, headache, fatigue, cognitive deficits, alterations in mood, and numerous multi-system complaints. Most potential exposures implicated in GWI were not well documented but included varying levels of several neurotoxicants as well as the anticholinergic drug pyridostigmine bromide (PB), which was routinely taken as prophylaxis against the nerve agent soman. While some veterans also took chloroquine as an antimalarial agent, the literature suggests an association between receipt of multiple vaccinations prior to or during the conflict (perhaps combined with other exposures), and GWI. In-theater exposures may account for any single individual veteran’s ill health but many veterans of the same era who were not deployed overseas also suffer the same or similar symptoms. The features of GWI also overlap with those of fibromyalgia, chronic fatigue syndrome and multiple chemical sensitivity, in all of which liver dysfunction has been documented, suggesting a unifying hypothesis. It is proposed that multiple vaccinations, with concurrent or subsequent exposure to PB or additional chemical insults of a liver-damaging nature, plausibly explain the pathogenesis and the observed chronicity of GWI. The suggested mechanism for GWI is thus a chemically-induced impaired liver function, with the spillage of stored vitamin A compounds (“retinoids”) into the circulation in toxic concentrations, resulting in an endogenous chronic form of hypervitaminosis A. Implications of the hypothesis are briefly reviewed.

## 1. Background

The 1990–1991 Gulf War began in response to the invasion and annexation of Kuwait by Iraq. The U.S.-led coalition of forces included the United Kingdom, Australia, France and Saudi Arabia, among others. By January 1991, coalition troops numbered 750,000, including 540,000 U.S. personnel. By the end of June 1991, the last U.S. troops had returned home. Soon after, veterans began to report chronic, unexplained health problems that did not fit established medical diagnoses and could not be explained by standard laboratory tests. An estimated 25% to 32% of the nearly 700,000 troops deployed to the theater of operations remain afflicted with chronic unexplained health complaints that have come to be known as Gulf War Illness (GWI). Many veterans of the same era who were not deployed overseas also suffer the same or similar symptoms. These multiple signs and symptoms include persistent musculoskeletal pain, fatigue, headache, difficulties with thinking, concentration and memory, irritable bowel syndrome, respiratory, dermatological and gastrointestinal complaints, sleep disturbances, sexual/reproductive difficulties, anxiety, and depression [1,2].

Numerous cohorts of Gulf War veterans have been assembled for scientific research purposes, including the large nationally representative Veterans Administration (VA) National Health Survey of Gulf War Veterans and Their Families [3,4]; the Iowa Persian Gulf Study Group [5]; studies of U.S. veterans from Oregon, Washington and Kansas; a study of Canadian Gulf War veterans; and studies of over 53,000 U.K. military personnel who served in the Gulf War [1].

The *National Health Survey of Gulf War Veterans and Their Families* began in 1995. This was a retrospective study, designed to be representative of those sent to the Persian Gulf and the 800,680 veterans who were not deployed but served in the military between September 1990 and May 1991. The VA mailed questionnaires to a stratified random sample of 15,000 Gulf War and 15,000 non-deployed veterans identified by the Defense Manpower Data Center (DMDC) [3]. The self-administered structured health questionnaire contained a 48-symptom inventory and questions about chronic medical conditions, functional limitations, use of medical services, and environmental exposures that included vaccinations, use of anti-nerve agent pyridostigmine bromide (PB) pills, smoke from oil-well fires, pesticides, and insecticides. In this major study, there was a higher prevalence of numerous unexplained health issues and of poor overall health in deployed veterans than in their nondeployed counterparts [3,4]. However, reports reviewed by the Institute of Medicine [1] found no association between being deployed to the Gulf War and specific health effects, nor between specific exposures occurring during deployment and health effects (e.g., diffuse pain such as headaches or musculoskeletal pain, fatigue, depression, cognitive impairments, respiratory, gastrointestinal, dermatologic and reproductive complaints). While Gulf War veterans consistently reported higher rates of nearly all symptoms examined than the nondeployed, these ailments were also seen to a lesser extent in Gulf Era veterans who were not deployed to the Gulf [1]. This was true whether the veterans were from the U.S., the U.K., Canada, Australia, or Denmark. An earlier report by the Institute of Medicine [6] additionally noted the characteristic multisystem nature of GWI; its overlap with fibromyalgia, chronic fatigue syndrome (CFS), and multiple chemical sensitivity (MCS); increased rates of amyotrophic lateral sclerosis and post-traumatic stress disorder; and increased risks of birth defects in the children of Gulf War veterans.

A follow-up study of the National Health Survey of Gulf War Veterans and their Families cohort in 2012 continued to report a higher prevalence of almost all queried physical and mental health conditions among Gulf War than Gulf Era veterans. Yet more than 20 years after the conflict, both Gulf War and Gulf Era veterans continue to report poor health (see Table 1 below) [7]. 

## 2. Potential Exposures

Although little is known about most Gulf War exposures, in part because medical procedures provided to veterans prior to and during deployment were not well documented [7], the IOM [1] found insufficient evidence to determine the existence of an association between GWI and any specific battlefield exposure or exposures. In particular, as noted in the 2010 IOM report [1] (p. 109), “The excess of unexplained medical symptoms reported by deployed GW veterans cannot be reliably ascribed to any known psychiatric disorder.” Stress and related psychiatric etiologies have thus been ruled out [8]. 

*Neurotoxic chemicals* were thought to be implicated, as nervous system symptoms are prominent and many neurotoxicants were present in theater, including organophosphates (OPs), carbamates and other pesticides, nerve agents, and pyridostigmine bromide (PB, anti-nerve agent) pills taken as prophylaxis against chemical warfare. The Research Advisory Committee on Gulf War Veterans’ Illnesses (RAC) concluded that GWI probably resulted from exposure to PB, pesticides, and possibly other factors including multiple vaccinations (see below) [9].

*Antimalarials*—It is well known that the quinoline class of antimalaria drugs has the potential to induce neuropsychiatric effects [10]. Based on the contemporary personal experience of one of us (AC), chloroquine was widely but erratically used by some units of deployed British and U.S. troops in the early weeks of the 1991 Gulf War; other units and individuals took doxycycline, a drug from the tetracycline class, and others took no antimalaria drugs. It remains plausible that the use of chloroquine and other quinoline antimalarials could have been a contributory factor in the evolution of GWI, which affected some deployed troops but not others, and was reported by British and U.S. veterans but not by French veterans of the conflict, who were never prescribed antimalaria drugs (AC, contemporary personal experience). To date, there is no evidence that antimalaria drugs played a role in GWI [11]. 

*Multiple Vaccinations*—The literature suggests that one of the factors most consistently associated with GWI was the reported number of administered vaccinations; that is, the more the number of vaccinations received, the greater was the likelihood of GWI. Both Gulf War and Gulf Era (nondeployed) veterans received the standard series of vaccinations against infectious diseases provided to any U.S. citizen traveling to the Gulf. These included yellow fever, typhoid, cholera, hepatitis B, meningitis, pertussis, polio, and tetanus. Anthrax vaccine was also administered to 150,000 troops and Botulinum toxoid to about 8000 troops [12]. At the time of the Gulf War, new recruits received up to 17 antigens during the first two weeks of basic training (see Table 2) [9].

Nearly all Gulf War veterans reported getting at least one vaccine for deployment: 70% reported receiving >5 vaccines and 30% received >10. Studies increasingly link GWI to the administration of multiple vaccinations and the constituents of vaccines, as documented below [9]:

*Unwin et al*. [13]—A postal survey was carried out on a random sample of Gulf War veterans (n = 4248), servicemen deployed to the Bosnia conflict (n = 4250, stratified for age and rank), and those serving during the 1991 Gulf War but not deployed there (Era cohort, n = 4246), regarding exposures, symptoms, and illnesses. The analysis was restricted to males. Measured outcomes were physical health, functional capacity (Short Form-36), the General Health Questionnaire, the Centers for Disease Control and Prevention (CDC) multisymptom criteria for Gulf War illness (GWI), and post-traumatic stress reactions. The Gulf War cohort reported symptoms and disorders significantly more frequently than the Bosnia and Era cohorts, the latter of which were similar. Gulf War veterans were more likely to report substantial fatigue and symptoms of stress, and nearly twice as likely to meet the CDC criteria for GWI as the other cohorts (Odds Ratio 2.5; 95% Confidence Interval: 2.2, 2.8). The CDC criteria were met by 61.9%, 36.8%, and 36.4% of the Gulf War, Bosnia, and Era cohorts, respectively. Although the analysis did not control for other potential exposures, it was reported that “vaccination against biological warfare and multiple routine vaccinations were associated with GWI in the Gulf War cohort.”

*Steele* [14]—To determine whether Kansas Gulf War veterans were affected by excess health problems, a population-based survey was conducted in 1998 on 1548 veterans who served in the 1991 Gulf War (GW) and 482 veterans who served elsewhere (non-GW). GWI occurred in 34% of GW veterans and 12% of non-GW veterans who reported receiving vaccines during the war, but only in 4% of non-GW veterans who did not receive vaccines. Non-GW veterans who reported being vaccinated during that time had significantly higher rates of chronic somatic pain, neurological, and gastrointestinal problems, and a nearly four-fold higher rate of GWI than non-GW veterans who did not receive vaccines. However, the rate of GWI in veterans who served in theater, all of whom were vaccinated, was 11 times higher than among non-deployed veterans who did not receive vaccines. These data strongly suggest that vaccines used during the war were a contributing factor to the excess morbidity among deployed and non-deployed Gulf War veterans.

*Hotopf et al.* [15]—A follow-up British study on that of Unwin et al. [13] explored the association between ill health after the war and vaccines received before or during the conflict. The hypothesis tested was that such ill health was limited to military personnel who received multiple vaccines during deployment and that pesticide use modified any effect. Gulf war veterans in the U.K. Armed Forces who still had their vaccine records were followed for six to eight years after deployment. Multisymptom illness was classified in terms of six features: fatigue; psychological distress; post-traumatic stress reaction; health perception; and physical functioning. The response rate for the survey was 70.4% (n = 3284), of which 28% (923) had vaccination records. Receipt of multiple vaccines *before* deployment was associated with post-traumatic stress reaction, whereas receipt of multiple vaccines *during* deployment was associated with all five of the other outcomes. Multiple vaccinations were associated with a 5.0-fold increased odds of multisymptom illness (95% CI: 2.5, 9.8). As in the earlier study by Unwin et al. [13], these results suggested a strong association between receipt of multiple vaccines and GWI. 

*Cherry et al.* [16]—In a study of personnel who served with the U.K. forces in the Gulf war, self-reported exposures were assessed in relation to symptoms via responses to symptom and exposure questionnaires completed seven or more years after the war. Participants included 7971 personnel who had been deployed in the Gulf from two exposed cohorts, with an overall response rate of 85.5%. The numbers of vaccinations, days handling pesticides, and days exposed to smoke from oil fires were consistently and independently related to symptom severity. Vaccinations were also significantly associated with the total number of days veterans reported taking PB pills. The number of vaccinations received was also associated with higher symptom scores on skin and musculoskeletal complaints. Relations between exposures and ill health were generally weak. However, “consistent, specific, and credible relations warranting further investigation were found between health indices and both the reported number of inoculations and days handling pesticides.” 

*Kelsall et al.* [11]—In a postal questionnaire-based study of 1456 Australian Gulf War veterans and a comparison group who were in operational units at the time of the war but were not deployed to the conflict (n = 1588), self-reported medical conditions were assessed and rated by a physician. Participants were asked to use their Immunization Booklet to report the number of vaccinations received. Gulf War veterans had a higher prevalence of all self-reported health symptoms than the control group, and more of the Gulf War veterans had severe symptoms. The total number of health symptoms reported in the past month by GW veterans was associated with increasing numbers of vaccinations, i.e., a dose-response relationship. The total number of symptoms was associated with having at least 10 vaccinations, but not with having any vaccination or with having a cluster of vaccinations (that is, >5 vaccinations within one week). The total number of self-reported symptoms in the past month was also associated with other Gulf War exposures, including taking PB pills or other anti-biological warfare tablets, exposure to pesticides, using insect repellants, and reportedly being in a chemical weapons area. It was noted that the association between receipt of 10 or more vaccinations and increased symptom reporting in GW veterans was consistent with previous studies, e.g., Unwin et al. [13], Hotopf et al. [15] and Cherry et al. [16]. Overall, a decade or more after the 1991 Gulf War, Australian veterans reported all symptoms and some medical conditions more commonly than those in the comparison group. 

The suggestion that multiple vaccinations may have been a key factor in GWI rather than exposures specific to deployment is supported by the following observations: French forces deployed to the Gulf war neither developed GWI nor received multiple vaccinations against biological warfare agents [17], but may not have been exposed to other GWI-related factors. GWI developed among non-deployed U.S. troops who received multiple vaccinations [1]. GWI-like syndromes were also reported by U.K. veterans of the Bosnia campaign of 1994–1995, all of whom received the required vaccinations [13]. 

The Research Advisory Committee’s report [9] (p. 20) on GWI and vaccinations concluded that although little information was available on the short- or long-term adverse effects of specific combinations of vaccines or the large number of vaccines received concurrently, the evidence suggested that receipt of multiple vaccinations contributed to the development of chronic symptoms in U.S. Gulf War era veterans. The U.K. studies likewise reported associations between receipt of multiple vaccinations and GWI, but found no interactive or synergistic effects between PB and receipt of multiple vaccines on any of the GWI parameters studied. The RAC report ([9], pp. 122–123) also noted that little reliable evidence was available that anthrax vaccine was a cause of GWI. It was rather the receipt of multiple vaccinations together than any single vaccine alone that appeared to contribute to GWI. Thus Unwin et al. [13] found that British Gulf War veterans who received the largest number of vaccines for the war had significantly worse health outcomes than those who received fewer vaccines. In the follow-up study by Hotopf et al. [15], among the 923 British veterans with vaccination records, the overall association between multiple vaccines and ill health was not specific to post-deployment vaccines [15]. Among Australian Gulf War veterans, those who reported higher symptom rates received the largest number of vaccinations [11]. In a second British study by Cherry et al. [16] that controlled for the effects of multiple exposures during deployment, the number of vaccinations received by veterans remained significantly associated with overall symptom severity and symptoms of peripheral neuropathy; there was also no difference between the effects of vaccines received before deployment and during the course of deployment [16]. Thus, while the types of vaccines and combinations of vaccines and their ingredients linked to GWI remain unknown, the literature suggests that it was the number of vaccinations received rather than any particular type or combination of vaccines that was the key factor linking vaccination with GWI. 

The multiple-vaccination hypothesis of GWI is further supported by the observation in the Kansas study that Gulf War-era veterans who were not deployed but were vaccinated during that time had significantly higher rates of chronic somatic pain, neurological, and gastrointestinal problems, and a nearly four-fold higher odds of GWI than nondeployed veterans who did not receive vaccines; furthermore, the rate of GWI among veterans who served in theater was 11 times higher than that of nondeployed veterans who received no vaccines [14].

## 3. Multiple Vaccinations as the Initiating Cause of GWI

An assumption of mass vaccination programs is that the effects of vaccines are limited to the prevention of each targeted disease. However, common vaccines have been shown to have non-specific effects on health; that is, effects in addition to prevention of targeted infections [18]. These effects depend partly on the order in which vaccines are administered; in some cases the outcomes are beneficial whereas in others they can be harmful [19]. For instance, a recent retrospective study determined the health impact of diphtheria-tetanus-pertussis (DTP) and oral polio vaccine (OPV) in an area of Guinea-Bissau in the early 1980s. The child population had been followed-up nutritionally every three months and both vaccines were offered from 3 months of age at these follow-up sessions. Children were allocated by birthdate in what amounted to a “natural experiment”; that is, receiving the vaccinations early or late between 3 and 5 months of age. Mortality rates in children who received DTP at ages 3–5 months were compared to those of children who had not yet received the vaccine. Among the children who had received DTP only, the mortality hazard ratio (HR) was 10.0 (95% CI: 2.61, 38.6), whereas among those who had received both DTP and OPV vaccines the HR was 5.00 (95% CI: 1.53, 16.3). These retrospective results suggested that DTP vaccination carries high risks of death compared to being unvaccinated, but that OPV could modify the negative effect of DTP. As noted by the authors, DTP is the most widely used vaccine and the proportion receiving it is used as an indicator of the performance of national vaccination programs. Yet the available evidence suggests that DPT may harm more children from other causes than it saves from diphtheria, tetanus or pertussis [20].

The current US childhood vaccination schedule has been expanded and accelerated in recent decades, but the long-term health outcomes of vaccination have remained unknown. To address this question, the first author and colleagues carried out a pilot study to compare vaccinated and unvaccinated homeschool children (children educated at home) on a broad range of health outcomes [21,22]. The study was based on an anonymous online survey of mothers of 6–12-year-olds in four U.S. states: Florida, Louisiana, Mississippi and Oregon, yielding a sample of 666 children, among whom 261 (39%) were unvaccinated. The vaccinated were, as expected, significantly less likely than the unvaccinated to have been diagnosed by a physician with chickenpox and whooping cough, but unexpectedly were significantly more likely to have been diagnosed with allergic rhinitis (OR 30.1; 95% CI: 4.1, 219.3), eczema (OR 2.9; 95% CI: 1.4, 6.1), otitis media (OR 3.8; 95% CI: 2.1, 6.6), pneumonia (OR 5.9; 95% CI: 1.8, 19.7), and a neurodevelopmental disorder (NDD) (OR 3.7; 95% CI: 1.7, 7.9), defined as Attention Deficit Hyperactivity Disorder, Autism Spectrum Disorder, and/or a learning disability. The vaccinated were also significantly more likely than the unvaccinated to use medication for allergies (OR 21.5, 95% CI: 6.7, 68.9), to have used antibiotics in the past 12 months (OR 2.4, 95% CI: 1.6, 3.6), been fitted with ventilation ear tubes (3.0% vs. 0.4%, *p* = 0.018; OR 8.0, 95% CI: 1.0, 66.1), and spent one or more nights in a hospital (19.8% vs. 12.3%, *p* = 0.012; OR 1.8, 95% CI: 1.1, 2.7). Furthermore, supporting the possibility that the number of vaccinations received could be implicated in risks of chronic illness, it was found that partially vaccinated children had increased but intermediate odds of chronic disease between those of unvaccinated and fully vaccinated children, specifically for allergic rhinitis, Attention Deficit Hyperactivity Disorder, eczema, a learning disability, and NDD as a whole. In summary, although the cross-sectional design of the study limits causal interpretation, the strength and consistency of the findings, the apparent “dose-response” relationship between vaccination status (unvaccinated, partial and fully vaccinated) and several forms of chronic illness, and the significant association between vaccination and NDDs all support the possibility that some aspect of the current vaccination schedule could be contributing to risks of childhood morbidity. Nevertheless, the study findings should be interpreted with caution. First, additional research is needed to replicate the findings using larger samples and stronger research designs. Second, subject to replication of the findings, potentially detrimental factors associated with the vaccination schedule need to be identified and addressed and underlying mechanisms and susceptibility factors need to be better understood in order to optimize the impact of vaccination on children’s health. Thus, returning to our subject, it is tempting to infer from reviews of GWI and from this pilot study of U.S. homeschool children, as well as related studies, that receipt of multiple vaccinations may be similarly associated with increased risks of allergies, infections, and cognitive impairments.

## 4. Clues to the Pathobiology of GWI

*Mitochondrial Dysfunction*—Mitochondria are essential for generating adenosine triphosphate (ATP) and thus for normal cellular functions. Mitochondrial defects are known to occur in a variety of diseases, including cancer, some forms of heart disease, Alzheimer’s disease, and Parkinson’s disease, and characterize a number of rare genetic disorders in children whose manifestations range from muscle weakness to organ failure. A major aim of current research is to explain how mitochondrial defects lead to pathological states. Evidence of mitochondrial dysfunction caused by acetylcholinesterase inhibitors and resulting in mitochondrial impairment (with impaired transport of mitochondria via microtubules), has been reported in veterans with GWI compared to controls. This evidence was based on prolonged post-exercise recovery of phosphocreatine, a compound used as a backup energy store and a robust index of mitochondrial function [23].

*GWI overlaps with fibromyalgia, chronic fatigue syndrome, and multiple chemical sensitivity*, suggesting shared risk factors and mechanisms. Abnormal fatigue is one of the most prevalent and debilitating symptoms of GWI. Although the mechanisms are unknown, immune system abnormalities are suspected [6].

*Liver damage* is common to most if not all of these syndromes, as evidenced by increased liver enzymes and clinical diagnoses of liver disease [24,25,26,27]. In the study of Australian male Gulf War veterans [27], those with multisymptom illness were significantly more likely than the control groups to have been diagnosed with obstructive liver disease, defined as alkaline phosphatase >110 U/L and γ-glutamyl-transferase >60 U/L. Multisymptom illness in the deployed comparison group was also associated with inflammatory liver disease, defined as alanine aminotransferase >55 U/L and aspartate aminotransferase >45 U/L, and elevated random plasma glucose. Among the nondeployed (n = 1071), the odds of obstructive liver disease were 7.5-fold higher in those with multisymptom illness than in those without (95% CI: 2.01, 30.27).

There have also been regular reports from the Gulf War Veterans Information System (GWVIS) on claims for benefits, stating an increase in liver disease (https://www.va.gov/RAC-GWVI/docs/Gulf_War_Illnesses_Links/GWVIS_Aug_2008.pdf; and https://www.index.va.gov/search/va/va_search.jsp?SQ=www.benefits.va.gov&QT=GWVIS&RS=1).

## 5. Synthesis of the Research Literature: New Hypothesis

The precise mechanisms underlying links between multiple vaccinations, other exposures such as organophosphate pesticides, acetylcholinesterase inhibitors, liver damage and GWI await further study. Research has shown that many biological markers and processes—ranging from autonomic function to depressed NK cell number and activity, to increased autoimmune markers, to white matter abnormalities—are altered in GWI. The hypothesis presented here is offered as a unifying explanation for how a variety of chemical insults occurring near-simultaneously result in a common outcome: namely, a post-hepatic syndrome that presents with the characteristic symptoms and signs of GWI. As many investigators have found, liver enzymes (notably, serum aminotransferase and alkaline phosphatase) are commonly raised in GWI sufferers, but because these enzymes can arise adaptively in settings of oxidative stress that are in no way linked to military service or to Gulf War chemical exposures, they cannot be considered as pathognomonic of GWI, which is a diagnosis that must take into account the individual’s entire social, occupational and vaccination history. 

**Figure 1:**
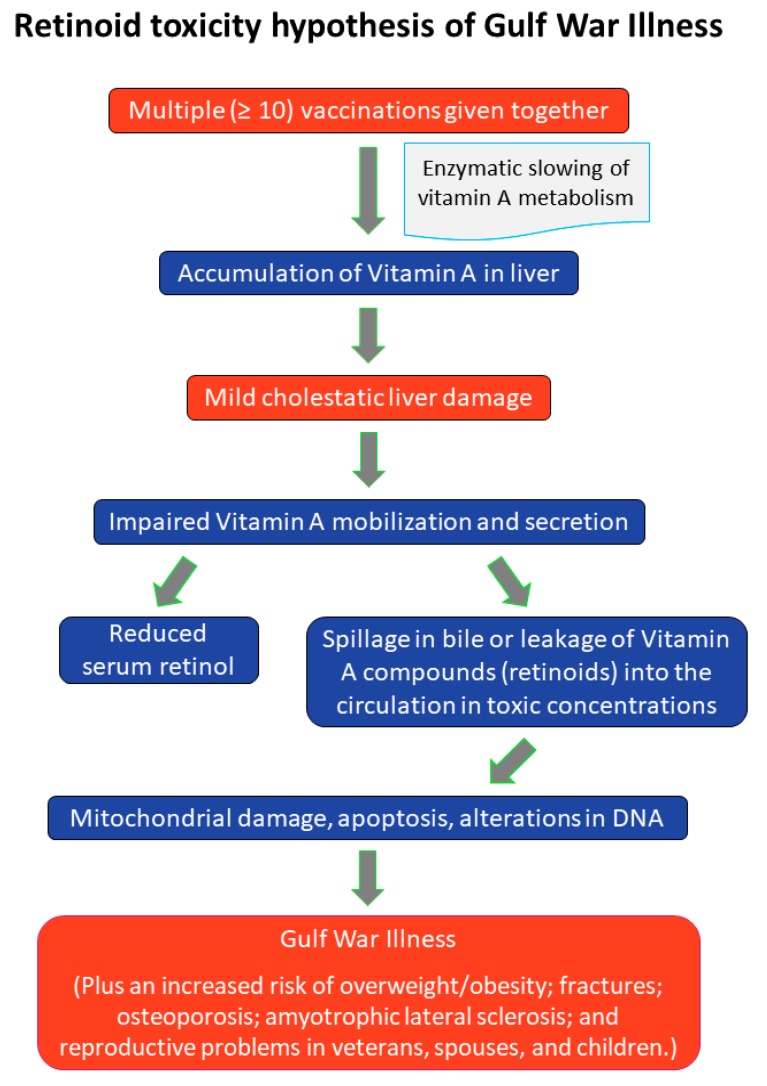
Retinoid toxicity hypothesis of Gulf War Illness.

Specifically, we propose that the adverse effects of multiple vaccinations received together, or over a short period of time, concurrently with other biochemical insults to the liver, may be initiated by interference with the hepatic metabolism of vitamin A, causing a mild cholestatic condition in which stored vitamin A metabolites (“retinoids”) spill over in the bile or leak into the circulation from damaged hepatocytes in toxic concentrations, inducing mitochondrial damage and apoptosis. On this hypothesis, the signs and symptoms of GWI as well as the related syndromes of fibromyalgia, chronic fatigue and multiple chemical sensitivity are manifestations of liver damage and a resulting chronic, endogenous form of hypervitaminosis A (see Figure 1).

Retinoids are fat-signaling molecules that derive mainly from dietary sources, and about 80% is stored in the liver in sufficient quantity to last the average adult for over 2 years without the need for additional intake. In normal physiological concentrations, retinoids are essential for multiple biologic functions, including cellular homeostasis, embryonic development, tissue differentiation and growth [28,29]. However, in higher concentration retinoids are cytotoxic, prooxidant, mutagenic and teratogenic. Retinoids are stored in the liver as retinyl esters and are normally secreted harmlessly into the circulation, as retinol-binding protein (RBP). If released unbound to protein, retinyl esters can be extremely toxic. An accepted indicator of retinoid toxicity is percent retinyl esters >10% of total vitamin A (retinol plus esters) [30,31]. The range of serum retinoic acid associated with acute or chronic vitamin A toxicity is not well defined. Serum retinol concentrations are normally 1–3 μmol/L but do not reflect hepatic vitamin A concentrations over a wide range of liver values, since the secreted RBP is under homeostatic control and serum levels can be low due to impaired hepatic mobilization and secretion.

**Figure 2:**
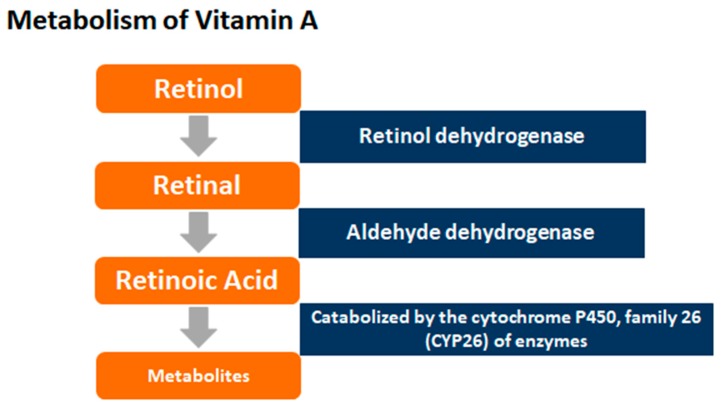
Metabolism of Vitamin A.

Retinoic acid (RA), the main biologically active form of vitamin A, binds to and activates specific retinoid receptors that regulate the transcription of many target genes [32]. RA is produced from free retinol via the hydrolysis of retinyl esters in the liver, the release of retinol into the circulation, and its subsequent delivery to the target tissues bound to retinol-binding protein (RBP). Retinoic acid is synthesized from the oxidation of retinol to retinaldehyde via an alcohol dehydrogenase, and from retinaldehyde via an aldehyde dehydrogenase reaction [33] (see Figure 2).

## 6. Review of the Evidence

Liver damage is associated with reduced serum retinol levels due to impaired hepatic mobilization, with increased liver enzyme levels [34]. Retinoid-associated hepatotoxicity leads to a form of cholestatic (obstructive) liver dysfunction in which vitamin A metabolites in bile regurgitate into the circulation, raising the level of all biliary substances in the blood [35,36]. Stored retinyl esters also leak from damaged hepatocytes [37]. The hypothesized net effect of these changes is a state of mild cholestasis and an endogenous form of hypervitaminosis A associated with an increased percentage of plasma retinyl esters as a fraction of total vitamin A, together with increased retinoic acid (RA) concentrations, and low or normal concentrations of retinol and its transporter, RBP. To date, there have been no studies of vitamin A metabolism and concentration profiles in GWI. Table 3 (below) shows that the diverse features of GWI closely mirror those of chronic hypervitaminosis A.

*Neuropsychiatric Symptoms and Mitochondrial Damage*—Therapeutic use of 13-cis-RA (isotretinoin) for acne is associated with the onset of depression, psychosis and suicide [44]. Therapeutic doses induce cognitive disturbances and depression in 1% to 11% of patients [45]. The neuropsychiatric symptoms of acute vitamin A poisoning include drowsiness, irritability, severe headaches, nausea, and various forms of impulsive and irrational behavior [46]. In mice, 13-cis-RA adversely affects learning and memory and significantly increases depression-like behaviors; acute and chronic vitamin A supplementation of laboratory animals at therapeutic doses also impairs mitochondrial function in liver, hippocampus and substantia nigra, decreases brain-derived neurotrophic factor levels and dopamine D2 receptor levels, decreases glutamate uptake, and alters locomotor and exploratory activity [45,47].

*Gastrointestinal*—Retinoids are linked to a wide range of adverse and often severe gastrointestinal effects [48,49].

*Pain/Headache/Migraine*—Severe headache (pseudotumor cerebri) and skeletal pain are major features of GWI and of hypervitaminosis A [50,51].

*Fatigue and Respiratory Problems*—Abnormal fatigue is a major feature of GWI and a defining feature of chronic fatigue syndrome as well as fibromyalgia and multiple chemical sensitivity [52,53]. Symptoms of severe fatigue and related signs of chronic hypervitaminosis A appear between 2–3 months and even years after excessive vitamin A exposure through dietary intake, supplementation or endogenous sources [54]. Treatment with synthetic retinoids can also cause fever and general fatigue, dyspnea, and respiratory distress, in some cases requiring endotracheal intubation and mechanical ventilation [54,55].

*Multiple Chemical Sensitivity*—Chemical sensitivity was reported by 28% of U.K. Gulf veterans and in 14% by those serving during the Gulf war but who were not deployed [53]. Mast cells (MCs), best known for their role as effectors of IgE-mediated allergic reactions, are increased in patients with symptoms of pruritus and in atopic dermatitis, psoriasis and urticaria [56], and express high levels of the retinoid receptor subtype Retinoic Acid Receptor-alpha [57]. RA also interferes with proliferation of skin MCs and promotes MC degranulation [58], supporting the concept that RA has a pro-allergic and pro-inflammatory-maintaining function in skin MCs [59]. 

*Bone Fractures*—Gulf war veterans suffered disproportionately from hospitalizations for fractures as well as bone and soft tissue injuries compared to nondeployed veterans of the same era [60]. High intakes of vitamin A and blood retinol levels are associated with increased risks of hip fracture [61]. 

*Amyotrophic Lateral Sclerosis (ALS)*—ALS has been linked to GWI. Deployed veterans were almost 2-fold more likely than the non-deployed to have been diagnosed with ALS in the early years after the war [62]. Mice with the mutated gene for Cu/Zn superoxide dismutase (G93A) are a good model for ALS, developing progressive limb paralysis with loss of motor neurons of the cervical and lumbar spinal cord around 3 months of age, and die within 4–5 months. A potential role for retinoid toxicity is suggested by the observation that long-term dietary supplementation with RA significantly shortened the lifespan of G93A mice but appeared to have no effect on motor neurons in the spinal cord [63].

*Reproductive disorders*—In the Veterans Administration’s large national survey, Gulf war veterans reported significant excesses of birth defects among their liveborn infants, including increased rates of Goldenhar Syndrome (involving deformities of the face) [64], as well as higher rates of spontaneous abortion and stillbirth [65]. Vitamin A and its derivative retinoic acid play essential roles in early embryonic patterning and organogenesis in vertebrates, and congenital malformations can result from exposure to excessive or decreased embryonic RA levels [66,67]. Multiple vaccinations and resulting liver damage and the release of toxic concentrations of stored vitamin A compounds into the maternal and fetal circulation could thus be the mechanism of the reproductive disorders reported in excess by GW veterans.

## 7. Discussion and Conclusions

Gulf War Illness (GWI) continues to cause distress and disability in about a third of veterans of the 1990–1991 Gulf war, affecting their spouses and children in complex ways. The conclusion of the latest report by the Institute of Medicine [68] on the health effects of serving in the Gulf War was that it was caused by post-traumatic stress disorder and resulted in multisymptom illness, but how this experience led to GWI remains uncertain. 

Another concern regarding GWI has been the potential long-term adverse effects of exposure to open burn pits. The Institute of Medicine’s 2011 report on the subject, however, found inadequate or insufficient evidence of a relation between exposure to combustion products and cancer, other diseases, and adverse reproductive outcomes [69].

With recent findings suggesting immune-related alterations in brain communication in GWI [70], a neuroimmune model has been proposed [71] as a framework for research to explain the continuation or worsening of GWI and to identify biomarkers for the illness. The model would take into account potential differences in risk based on gender, on time elapsed since exposure to neurotoxicants, the duration and severity of illness, comorbid conditions, and genotype. In fact, GWI appears to be more common in female than in male veterans and is associated with excess rates of breast cysts, abnormal Papanicolaou smears, yeast and bladder infections, and significantly increased rates of adverse reproductive outcomes including birth defects [72].

An alternative model of the etiopathogenesis has been presented here, suggesting that GWI is a post-hepatic syndrome initiated by the receipt of multiple vaccinations and their ingredients administered synchronously or near-synchronously, possibly in combination with additional chemical insults to the liver, resulting in the entry of normally stored retinoids into the circulation in toxic concentrations. The model could thus explain the development of chronic symptoms both in deployed and nondeployed veterans from several countries. Further research is needed to evaluate and ensure the safety of vaccines requiring improved understanding of systems biology related to adverse events occurring after vaccines [73]. In individual cases, one or more concurrent chemical insults of a liver-damaging nature (neurotoxicants, PB, chloroquine, mefloquine) may also have contributed to the pathogenesis of GWI. For instance, evidence suggests that excess illness in Gulf War veterans could be explained in part by exposure to organophosphate and carbamate acetylcholinesterase inhibitors, including pyridostigmine bromide (PB), pesticides, and nerve agents [74].

The retinoid-toxicity hypothesis could be tested by comparing veterans with GWI and unaffected controls in terms of liver function, dehydrogenase enzyme activity, retinoid concentration profiles (retinol, retinyl esters, percent retinyl esters, and retinoic acid) and retinoid receptor expression. In addition to evidence of liver dysfunction, as previously documented [11], cases would be expected to have significantly reduced dehydrogenase enzyme activity, increased retinoic acid concentrations and receptor expression, low or normal concentrations of retinol and its transporter, RBP, and an increased percentage of plasma retinyl esters as a fraction of total vitamin A, after adjustment for other potential exposures and mechanisms. 

The hypothesis suggests that current treatment strategies for GWI should incorporate the strict avoidance of all liver-damaging substances, including alcohol and prescription drugs that are metabolized in the liver. The dietary intake of rich sources of vitamin A such as milk, cheese, egg yolks and fish oils [75] could also be avoided or much reduced. With regard to health policy, the practice of administering multiple vaccinations simultaneously, or within a highly compressed time frame, should be subject to an urgent safety review. Research is needed to evaluate the health effects of mass vaccination programs, especially when (as here) the recipients are in a position of limited autonomy, and to understand the factors affecting individual responses to vaccination. The benefits derived from short-term protection against infectious disease also need to be weighed against the potential long-term health risks of multiple vaccinations administered synchronously or near-synchronously, and in association with other potentially toxic exposures.

Many unanswered questions remain about the risk factors and pathogenesis of GWI. Prior studies were also prone to a variety of epidemiologic biases (e.g., selection, surveillance, recall, immortal-time, protopathic). It is hoped that our hypothesis will stimulate further research to support or refute a causal association between multiple vaccinations, alterations in retinoid metabolism, and GWI.

## Figures and Tables

**Table 1:** Medical conditions associated with Gulf War Illness.

Condition	GW Veterans vs. Gulf Era Veterans (% Difference)	Adjusted Odds Ratio and 95% Confidence Interval
Chronic multisymptom illness	43.9% vs. 20.3%	2.36; 1.94, 2.86
Chronic fatigue syndrome	11.8% vs. 5.3%	2.36; 1.94, 2.86
Neuralgia	9.4% vs. 6.3%	1.65; 1.40, 1.95
Gastritis	20.2% vs. 14.3%	1.59; 1.35, 1.73
Chronic obstructive pulmonary disease	8.4% vs. 6.3%	1.48; 1.23, 1.78
Fibromyalgia	3.7% vs. 2.9%	1.48; 1.15, 1.91
Tachycardia	8.1% vs. 5.9%	1.47; 1.20, 1.79
Dermatitis	27.4% vs. 21.1%	1.44; 1.27, 1.63
Rheumatoid arthritis	9.9% vs. 7.9%	1.40; 1.17, 1.67
Seizures	2.7% vs. 2.0%	1.38; 1.03, 1.85
Coronary heart disease	5.6% vs. 5.3%	1.32; 1.09, 1.59
Migraine headaches	20.3% vs. 16.1%	1.30; 1.15, 1.47
Hypertension	43.0% vs. 40.0%	1.22; 1.10, 1.35
Asthma	10.2% vs. 9.0%	1.22; 1.04, 1.44
Unspecified arthritis	33.9% vs. 31.8%	1.16; 1.05, 1.29
Irritable bowel syndrome	24.4% vs. 14.3%	2.10; 1.79, 2.45
Functional dyspepsia:	27.7% vs. 15.9%	1.94; 1.75, 2.17
PTSD in the past 4 weeks	20.9% vs. 11.5%	1.93; 1.67, 2.24
Major depressive disorder, past 2 weeks	32.9% vs. 22.9%	1.56; 1.41, 1.73
Other depressive disorder, past 2 weeks	23.5% vs. 19.1%	1.24; 1.08, 1.38
Other anxiety disorder, past 4 weeks	18.7% vs. 14.4%	1.34; 1.17, 1.54
Somatic symptom severity, past 4 weeks	16.1% vs. 8.3%	2.10; 1.79, 2.45

Source: Ref. [7].

**Table 2:** U.S. Military Personnel Directed to Receive Vaccine Schedule at the time of the Gulf War.

Vaccine	Military Personnel	Schedule/Dose
Adenovirus	All recruits	1 oral dose
Influenza	All recruits and active duty	Annual shot
Measles	All recruits	1 shot
Meningococcal	All recruits, active duty as required	1st shot, then booster every 3–5 years
Plague	All Marines; Army and Navy special forces, others in at-risk occupations or deploying to high risk areas	5 shots over 12 months, then booster every 1–2 years
Polio	All recruits	1 oral dose
Rabies	Special forces, at-risk occupations	3 shot series
Rubella	All recruits	1 shot
Smallpox vaccine or booster	New recruits through the late 1980s	1 dose
Tetanus-diphtheria	All recruits, active duty, and reserve	Booster every 10 years
Typhoid	Army and Air Force alert forces and for deployment to high risk areas	2 doses in 2 months, then booster every 3 years
Yellow fever	All Navy and Marine Corps, Army and Air Force alert forces and for deployment to high risk areas	1st shot, then booster every 10 years

Source: Ref. [9].

**Table 3:** Similarities between the signs and symptoms of Gulf War Illness and of chronic hypervitaminosis A.

Major Signs/Symptoms	Gulf War Illness	Chronic Hypervitaminosis A
Mood swings, irritability	+	+
Memory loss, lack of concentration	+	+
Anxiety, stress, sleep disturbance	+	+
Depression	+	+
Other psychiatric disorders	+	+
Muscular pain, weakness	+	+
General Fatigue	+	+
Fevers/Night sweats	+	+
Headaches	+	+
Numbness, tingling, dizziness	+	+
Sinus congestion	+	+
Chronic, frequent infection	+	+
Skin allergies, other allergies	+	+
Respiratory problems	+	+
Digestive, stomach and intestinal disorders	+	+
Weight gain/loss	+	+
Reproductive disorders	+	+

**Sources:** GWI: [9,38,39,40]; Chronic hypervitaminosis A: [31,41,42,43].
